# Primary Congenital Glaucoma among the Children Under 3 Years of Age in the Outpatient Department in a Tertiary Care Hospital: A Descriptive Cross-sectional Study

**DOI:** 10.31729/jnma.5889

**Published:** 2021-09-30

**Authors:** Rakshya Panta Sitoula, Jamuna Gurung, Afaque Anwar

**Affiliations:** 1 Department of Glaucoma, Biratnagar Eye Hospital, Biratnagar, Nepal; 2 Department of ophthalmology, Gandaki Medical College and Teaching Hospital, Pokhara, Nepal; 3 Department of Medical Education, Biratnagar Eye Hospital, Biratnagar, Nepal

**Keywords:** *buphthalmos*, *intraocular pressure*, *primary congenital glaucoma*, *trabeculectomy*, *trabeculotomy*

## Abstract

**Introduction:**

Primary congenital glaucoma is a rare vision-threatening condition of children. Primary congenital glaucoma though a rare disease it is the most common cause of childhood glaucoma with potency to cause blindness. This study was undertaken to find the prevalence of the children with primary congenital glaucoma under 3 years of age in a tertiary care hospital.

**Methods:**

This was a descriptive cross-sectional study conducted at a tertiary eye center in Nepal in children (≤ 3 years) presented in the outpatient department of a tertiary eye hospital between June 2017 and June 2020. The study was approved by the hospital review committee and adhered to the declaration of Helsinki. A convenient sampling method was used. Point estimate at 90% Confidence Interval was calculated with frequency distribution. Data analysis was conducted using Statistical Package for the Social Sciences.

**Results:**

Out of total children under 3 years who presented to the outpatient department, 46 (0.31%) at 90% Confidence Interval (0.303-0.316) had primary congenital glaucoma. Among them, 30 children (65.2%) had bilateral involvement. Mean intraocular pressure was 42.40±8.15mm Hg. The mean age of initial presentation, horizontal corneal diameter, and axial length were 12.07±8.9 months, 12.95±1mm, and 23.89±1.7mm respectively. Consanguinity was observed in 12 (26%) children.

**Conclusions:**

From the study, we conclude that there was a low prevalence of primary congenital glaucoma among children under 3 years of age who presented to the outpatient department in a tertiary care hospital.

## INTRODUCTION

Primary congenital glaucoma (PCG) is a developmental anomaly of anterior chamber angle leading to the increase in intraocular pressure (IOP), corneal edema, Habb striae, increased corneal diameter, buphthalmos, and optic nerve damage.^1^ PCG is common in certain ethnic and religious groups where parental consanguinity is prevalent.^2-3^ The incidence of PCG is reported to be 1 in 10,000-20,000,1 in 3300, 1 in 3030, 1 in 2500, 1 in 1200 live births in the western world,^4^ Southern India^5^, Saudi Arabia^6^, Middle East^7^ and Slovakian Gypsies^8^ respectively.

PCG though a rare disease it is the most common cause of childhood glaucoma with potency to cause blindness. Goniotomy,^9,10^ trabeculotomy,^11,12^ and combined trabeculectomy with trabeculectomy have proven to be highly successful treatment modalities.^13-17^

There are lacunae in the literature addressing the scenario of children with congenital glaucoma in Nepal.

This study was undertaken to find the prevalence of the children with primary congenital glaucoma under 3 years of age in a tertiary care hospital.

## METHODS

This was a descriptive cross-sectional study conducted at a tertiary care ophthalmology center. Data were collected from the hospital record of all children ≤ 3 years of age that presented to OPD between June 2017 and June 2020. The study was approved by the Ethical review committee of the hospital, and adhered to the declaration of Helsinki. All the children below 3 having complete data were included in this study. Children with incomplete clinical records were excluded from the study. Vision in these children is difficult to assess as most of them were preverbal so visual acquity was not included in the study. A convenient sampling method was used.

The sample size was calculated by using formula:

n = Z^2^ × p × q / e^2^

  = (1.645)^2^ × (0.5)(1-0.5) / (0.01)^2^

  = 6765

Where,

n = required sample size
Z = 1.645 at 90% Confidence Interval
p = prevalence required for maximal sample size, 50%
q = 1-p
e = margin of error, 1%

Here the calculated sample size is 6765. After doubling the calculated sample size, the new sample becomes 13530. However, we have included 14614 children under 3 years visiting the outpatient department of a tertiary eye hospital for the study. Diagnosis of primary congenital glaucoma was made only after examination under anesthesia (EUA) as per guidelines given by Congenital Glaucoma Research Network (CGRN) guideline.^18^

All the records of the patients are filed in the glaucoma department and patients are discharged with the discharge summary. So, the data was taken from the hospital sheets which included EUA findings. A detailed history and demographic findings obtained at the first visit were recorded. History included duration of symptom, antenatal history, family history and consanguinity. Consanguinity was defined by a relationship by blood where parents or grandparents were first- or second-degree cousins.

Clinical examination data records were obtained from the EUA record sheets. Anesthesia used was ketamine with spontaneous ventilation. IOP was measured using tonopen (Reichert Technologies, Depew, New York, USA). EUA records included corneal diameter, corneal findings, Haab striae, iris and anterior chamber abnormalities, lens anomalies, cup to disc ratio, and axial length. Fundus findings were recorded only if corneal cloudiness did not preclude the visibility. Combined trabeculotomy with trabeculectomy (CTT) was carried out in one eye in the same sitting after completing clinical examination under anesthesia. All these children underwent trabeculotomy with trabeculectomy as all these children had hazy cornea which made goniotomy difficult.

Informed consent for surgical intervention was taken from the parents prior to subjecting the child for EUA and surgical intervention. Postoperatively during subsequent follow up ocular findings, intraocular pressure, optic disc findings and axial length recorded. Additional Informed consent was taken from the parent of all the children whose data were collected. Data analysis was conducted using Statistical Package for the Social Sciences.

## RESULTS

Out of total 14614 children under 3 years, 46 (0.31%) at 90% Confidence Interval (0.303-0.316) children diagnosed with primary congenital glaucoma.

The mean IOP of all the children at baseline was 42.40±8.15 mm Hg. There were 31 (67.39%) male and 15 (32.6%) female children, the ratio being 2:1. Thirty children (65.2%) had bilateral involvement. Hence altogether there were 76 (82.06%) (n= 96) eyes that underwent Combined trabeculectomy and trabeculotomy whose data was analyzed.

**Table 1:** Patient demographics (76 eyes of 46 patients) (n= 46).

Demographics	n (%)
**Age (years)**	
<1	36 (79)
> 1	10 (21)
**Gender**	
Male	31 (67.39)
Female	15 (32.6)
**Laterality**	
Bilateral	30 (65.2)
Unilateral	16 (34.8)

Mean age of initial consultation was 12.07±8.9 months. Majority of the children presented within 1 year of age 36 (79%). Family history of Glaucoma was recorded in two children where their elder sibling had congenital glaucoma. Consanguinity was seen in 12 (27%) of the children.

Mean horizontal corneal diameter was 12.97±0.99mm (Table 2). The axial length at presentation was (23.89±1.79). It was not possible to evaluate the cup disc ratio in the entire child preoperatively due to corneal haziness. In the children where C/D ratio could be assessed the mean cup disc ratio was 0.7±0.22 preoperatively. All the diagnosed children with PCG after EUA underwent same operative procedure combined trabeculotomy with trabeculectomy under general anesthesia.

**Table 2:** Clinical features on initial examination.

Clinical features	Mean±SD
Corneal diameter (mm)	12.95±0.99
Age of initial consultation (months)	12.07±8.9
Preoperative IOP (mm Hg)	42.40±8.15
Initial axial length	23.89±1.79
Initial cup-disc ratio	0.7±0.22

Complete success (IOP ≤ 21mm Hg without additional medication) was achieved in 52 (68%) of the eye that underwent surgery at 1 year of follow up. Relative success was achieved IOP <21mm Hg with 1 topical antiglaucoma medication in 62 (81.5%) of the children.

## DISCUSSION

This information obtained after analyzing the hospital record of children presenting to Biratnagar Eye Hospital with congenital glaucoma was of enormous importance. As this hospital is the main referral center for all the children with congenital glaucoma in the eastern region of Nepal this study was successful in establishing the scenario of congenital glaucoma in this region. Further, the importance of this study is enhanced by the fact that there is no published data on the date of congenital glaucoma in this region. In our study the primary congenital glaucoma was present in 46 (0.39%) children under 3 years.

In our study congenital glaucoma was found to affect the male child more than female: 67.39% and 32.6% respectively. Similarly, various studies have shown male predominance.^3,4,19^ However, a recent study from Saudi Arabia showed an equal sex distribution.^6^ Majority of the children had bilateral disease (65.2%) which is in consistency with the previous literatures of 70% to 80%^1-4,6^

Mean age of initial consultation was 12 months similar to a multicentered study by Papadopoulos, et al. (11.3 months)^3^ but delayed as compared to a study by Alanazi, et al.^6^ where the mean age was 3.9 months. Majority of the children were brought by the parents within 1 year of age (79%) which is consistent with various literatures.^4,20^ However, only 27% (13/48) of our cohort presented within 6 months of age though most of the parents noticed the haziness of cornea since 1-2 month of birth. This delay in initial presentation may be due to lack of awareness about congenital glaucoma among the parents of these children. When inquired specifically they told that they were in conception that small infants are not operated unless they are 1 year of age. As time of surgical intervention is very crucial it is essential that general Ophthalmologist and general practitioners are aware of this condition. In some developing countries like Ethopia the children with congenital glaucoma from rural regions presented at as late as 3 years of age.^21^

Family history was positive in only 3/46 (6%) children which is in lower than the published literature^4,6,20,22^ which gives a range from 10% to 40%. All three children had first degree cousin with congenital glaucoma and history of consanguineous marriage was positive. Various literature has shown an association between the incidence of congenital glaucoma and consanguineous marriage.^1,2,6,23^ In our cohort rate of consanguineous marriage was 27%, and all these children belonged to one specific religious group where cousin-cousin marriage is practiced. Study from Saudi Arabia^6^ showed consanguineous marriage to be as high as 59% of the children. The British Infantile Glaucoma study^3^ found that 16% of their cohort was born of consanguineous marriage and all were of Asian origin (67% were Pakistanis). This evidence highlights the importance of community education about drawbacks of consanguineous marriage, genetic counseling of the couples and screening of siblings is recommended in communities where cousin - cousin marriage is practiced.

All eyes underwent single operation; CTT without Mitomycin C (MMC). We were able to achieve postoperative IOP of ≤ 21 mm Hg in 65% (78% with medication) of the children after CTT which is comparable to other literatures.^3,6,15,17^ CTT was chosen initially instead of goniotomy or trabeculotomy alone because many studies have shown that CTT has shown the better IOP control.^13-14,17,22^ especially with advanced disease severity.^24^

The limitations of the study are that it is a descriptive cross-sectional study with biases. We cannot calculate the prevalence of congenital glaucoma in this region of Nepal as significant number of patients was from north-eastern regions of India. We addressed only PCG and had to exclude the SCG because of lack of hospital records of all these children. We cannot comment on long term results of the surgery.

## CONCLUSIONS

From the study, we conclude that there was a low prevelance of family congenital glaucoma among children under 3 years of age who presented to the outpatient department in a tertiary care hospatal.
